# Intranasal administration of trehalose reduces α-synuclein oligomers and accelerates α-synuclein aggregation

**DOI:** 10.1093/braincomms/fcae193

**Published:** 2024-08-20

**Authors:** Makoto T Tanaka, Yasuo Miki, Fumiaki Mori, Tomoya Kon, Tomonori Furukawa, Shuji Shimoyama, Yota Tatara, Taku Ozaki, Conceição Bettencourt, Thomas T Warner, Koichi Wakabayashi

**Affiliations:** Department of Neuropathology, Institute of Brain Science, Hirosaki University Graduate School of Medicine, Hirosaki 036-8562, Japan; Department of Neuropathology, Institute of Brain Science, Hirosaki University Graduate School of Medicine, Hirosaki 036-8562, Japan; Queen Square Brain Bank for Neurological Disorders, UCL Queen Square Institute of Neurology, London WC1N 1PJ, UK; Department of Neuropathology, Institute of Brain Science, Hirosaki University Graduate School of Medicine, Hirosaki 036-8562, Japan; Department of Neurology, Institute of Brain Science, Hirosaki University Graduate School of Medicine, Hirosaki 036-8562, Japan; Department of Neurophysiology, Institute of Brain Science, Hirosaki University Graduate School of Medicine, Hirosaki 036-8562, Japan; Department of Neurophysiology, Institute of Brain Science, Hirosaki University Graduate School of Medicine, Hirosaki 036-8562, Japan; Department of Stress Response Science, Hirosaki University Graduate School of Medicine, Hirosaki 036-8562, Japan; Department of Biological Science, Graduate School of Science and Engineering, Iwate University, Morioka 020-8551, Japan; Queen Square Brain Bank for Neurological Disorders, UCL Queen Square Institute of Neurology, London WC1N 1PJ, UK; Department of Neurodegenerative Disease, UCL Queen Square Institute of Neurology, University College London, London WC1N 3BG, UK; Queen Square Brain Bank for Neurological Disorders, UCL Queen Square Institute of Neurology, London WC1N 1PJ, UK; Department of Neurodegenerative Disease, UCL Queen Square Institute of Neurology, University College London, London WC1N 3BG, UK; Reta Lila Weston Institute of Neurological Studies, UCL Queen Square Institute of Neurology, London WC1N 3BG, UK; Department of Neuropathology, Institute of Brain Science, Hirosaki University Graduate School of Medicine, Hirosaki 036-8562, Japan

**Keywords:** multiple system atrophy, trehalose, intranasal administration, α-synuclein oligomer, aggregation

## Abstract

Abnormal α-synuclein (αSyn), including an oligomeric form of αSyn, accumulates and causes neuronal dysfunction in the brains of patients with multiple system atrophy. Neuroprotective drugs that target abnormal αSyn aggregation have not been developed for the treatment of multiple system atrophy. In addition, treating diseases at an early stage is crucial to halting the progress of neuronal damage in neurodegeneration. In this study, using early-stage multiple system atrophy mouse model and *in vitro* kinetic analysis, we investigated how intranasal and oral administration of trehalose can improve multiple system atrophy pathology and clinical symptoms. The multiple system atrophy model showed memory impairment at least four weeks after αSyn induction. Behavioural and physiological analyses showed that intranasal and oral administration of trehalose reversed memory impairments to near-normal levels. Notably, trehalose treatment reduced the amount of toxic αSyn and increased the aggregated form of αSyn in the multiple system atrophy model brain. *In vitro* kinetic analysis confirmed that trehalose accelerated the aggregate formation of αSyn. Based on our findings, we propose a novel strategy whereby accelerated αSyn aggregate formation leads to reduced exposure to toxic αSyn oligomers, particularly during the early phase of disease progression.

## Teaser

Tanaka *et al.* report that the intranasal administration of trehalose reduced toxic α-synuclein oligomers and increased α-synuclein aggregates in the hippocampus of a mouse model of human α-synuclein-inducible multiple system atrophy. This treatment may reduce exposure to toxic α-synuclein oligomers by accelerating the formation of aggregates and improve memory.

## Graphical Abstract

**Graphical Abstract:**
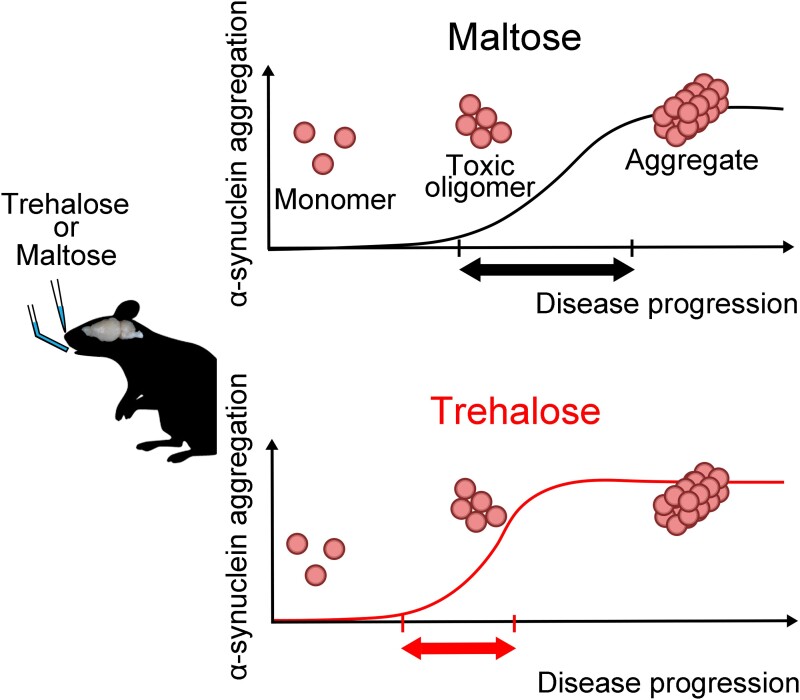

## Introduction

Lewy body diseases (Parkinson’s disease and dementia with Lewy bodies) and multiple system atrophy (MSA), collectively known as synucleinopathies, share the neuropathological feature of abnormal α-synuclein (αSyn) species accumulated in the central and peripheral nervous systems.^1,2^

Abnormal αSyn species are incorporated into pathologically specific inclusions, such as Lewy bodies in Lewy body diseases or glial cytoplasmic inclusions in MSA.^1,2^ These inclusions have been thought to be toxic to neurones, as areas of significant neuronal loss have a predilection for Lewy bodies.^3^ However, recent studies have suggested that the formation of inclusions may instead provide neuroprotection by segregating abnormal αSyn species from healthy organelles and acting as a platform for the degradation of abnormal αSyn by the autophagy-lysosome or ubiquitin-proteasome system.^3-9^

Eliminating or reducing the amount of toxic αSyn serves as a therapeutic strategy for synucleinopathies. Unfortunately, despite a growing number of studies aimed at developing interventions to activate key intracellular pathways for the degradation of abnormal proteins (autophagy-lysosome system) or to clear abnormal αSyn using anti-αSyn antibodies, no effective treatment has been developed to delay or prevent the onset and progression of synucleinopathies.^10,11^ Several factors may contribute to the limited progress in treating synucleinopathies, including the poorly understood molecular mechanisms of disease pathogenesis, and the limited delivery of drugs to the brain due to the blood–brain barrier. Several clinical trials have also shown that preclinical or early-stage intervention is crucial to halting the progress of neuronal damage in neurodegeneration.^12-16^ Recently, we found that trehalose intake seemed to activate autophagic flux in the brains of Lewy body disease model mice and controls.^17^ However, the oral administration of trehalose was not effective enough to clear abnormal αSyn aggregates from the brains of the model.^17^ Thus, using an early-stage mouse model, we focused on intranasal delivery, which can bypass the blood–brain barrier and reach the brain via the olfactory nerve in the olfactory epithelium and/or the trigeminal nerve in the respiratory epithelium.^18,19^

To this end, we generated a mouse model of human αSyn-inducible MSA that displays some of the pathological and biochemical features of MSA, allowing therapeutic intervention shortly after αSyn accumulation in oligodendrocytes.^20,21^ Similarly to human MSA cases, MSA mouse model develops abnormal human αSyn in various brain regions including the hippocampus, followed by memory impairment and motor symptoms four weeks and one year, respectively, after human αSyn induction.^20,21^ Using this model and human cases of MSA, we previously identified an oligomeric form of αSyn as the pathological substrate of cognitive impairment.^21,22^ In the present study, using this early stage of MSA mouse model, we investigated whether intranasal administration of trehalose serves as an effective strategy for the treatment of MSA.

## Materials and methods

### Animals

All animal experiments in the present study were performed in accordance with the guidelines for animal experiments and were approved by the Animal Research Committee of Hirosaki University (approval number: AE01-2023-164). A total of 71 mice (mean age 32.51 weeks) including MSA model mice and controls were used in the present study. The demographic data and product information of the present study are shown in the Supplementary Tables 1 and 2. To generate a MSA mouse model, we mated a human α-Syn-loxP transgenic mouse with a Cre recombinase (Cre)/estrogen receptor (ER) transgenic mouse, in which Cre/ER is driven by the mouse proteolipid protein 1 promotor.^20^ A schematic diagram of the inserted gene on αSyn-loxP transgenic mice is shown in Supplementary Fig. 1. Tamoxifen (100 mg/kg, intraperitoneally) was injected once per day for 5 days to activate Cre system. After tamoxifen injection, human αSyn is expressed in oligodendrocytes and forms glial cytoplasmic inclusion-like structures in the brain.^20^ In the present study, age- and gender-matched MSA model mice were orally and intranasally administered 2% (w/v) trehalose or maltose. In addition, the mice underwent intranasal administration every week (eight times for 60 days): a total of 10 μL of 2% trehalose or maltose in 1× phosphate buffered saline into the nasal cavity. In the present study, we divided the mice into four groups: oral maltose intake with intranasal maltose (MM) or trehalose (MT), and oral trehalose intake with intranasal maltose (TM) or trehalose (TT). It was previously shown that maltose has no effect on αSyn clearance.^17^ Therefore, maltose was used as a control in the present study. Disaccharides were purchased from Wako and dissolved in drinking water, which was changed every 2 days. A schema of the time course of the experimental design is shown in Supplementary Fig. 2.

### Analyses

All methods for the present study are detailed in the Supporting information; these include mass spectrometry, behavioural analysis, electrophysiological analysis, immunohistochemistry, immunoblotting, filter trap assay, proximity ligation assay (PLA), *in vitro* kinetic assay and quantitative analysis.

### Statistical analysis

All data are expressed as the mean ± standard deviation. All statistical analyses were performed using R (R4.3.1). After Shapiro–Wilk test, if *P* < 0.05, we performed Mann–Whitney U-test or Kruskal–Wallis followed by Steel-Dwass test. If *P* ≥ 0.05, we performed F test or Bartlett’s test. If *P* < 0.05 in F test, Welch two sample *t*-test was performed. If *P* ≥ 0.05 in F test or Bartlett’s test, Student *t*-test or one-way ANOVA followed by Tukey test were performed. *P*-values of <0.05 were considered statistically significant.

## Results

### Confirmation of trehalose delivery to mouse brain by intranasal administration

**Figure 1:**
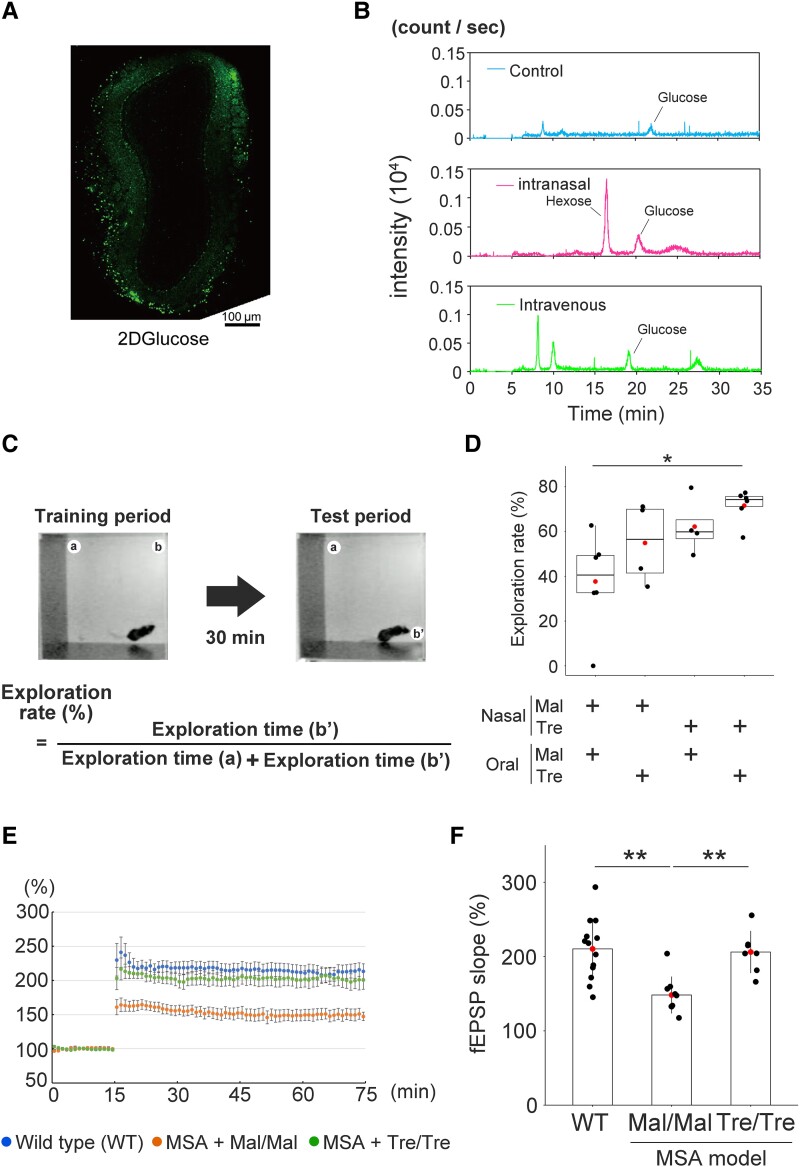
**Multiple system atrophy (MSA) model mice show memory impairment after human α-synuclein (αSyn) induction.** (**A**) Fluorescent signals are detected in the olfactory bulb 15 min after injection of fluorescent glucose (a total of 10 µL of 50 mM) into the nose of the control mice. (**B**) Liquid chromatography-tandem mass spectrometry showing the kinetics of trehalose in the mouse brain. Although trehalose is not detected as a peak in any of the groups (retention time 27.9 min), hexose is detected only in the brain of mice administered trehalose intranasally at a retention time of 16.4 min. (**C**) The schema of object-location memory task test. (**D**) We gave the water with 2% of trehalose (Tre) or maltose (Mal) to MSA model mice for two months, and intranasally administered 10 µL of 2% trehalose or maltose one per week for two months after tamoxifen injection. We used oral and intranasal maltose (MM or Mal/Mal) MSA group as a control in this study. After treatment finished, we performed behavioural analysis to assess the memory impairment. Behavioural analysis of the MSA model mice shows that intranasal and oral trehalose (TT or Tre/Tre) administration reversed memory impairment in the model mice (*n* = 6), whereas MM treatment had no such effect on cognitive function (*n* = 6). Additionally, oral maltose and nasal trehalose (MT) (*n* = 4), and oral trehalose and nasal maltose (TM) (*n* = 4) groups were unchanged relative to MM group. *P < 0.05. (**E**, **F**) Electrophysiological analysis shows that hippocampal long-term potentiation (LTP) in the CA1 region is significantly impaired in the MSA model mice [WT (*n* = 13) versus MM-MSA (*n* = 9), **P < 0.01]. However, this impairment is prevented by trehalose treatment [MM-MSA versus TT-MSA (*n* = 7), **P < 0.01]. We used Kruskal–Wallis followed by Steel-Dwass for **D**, and one-way ANOVA followed by Tukey–Kramer for **F**. Test statistic: (**D**) *z*-value: 1.0801234, *P*-value: 0.70170769 [MM-MSA, *n* = 6 versus MT-MSA, *n* = 4]; *z*-value: 2.0059435, *P*-value: 0.18569581 [MM-MSA versus TM-MSA, *n* = 4]; *z*-value: 3.0641294, *P*-value: 0.01171856 [MM-MSA versus TT-MSA, *n* = 6]; *z*-value: 0.5773503, *P*-value: 0.93889658 [MM-MSA versus TM-MSA]; *z*-value: 1.9188064, *P*-value: 0.22004150 [MT-MSA versus TT-MSA]; *z*-value: 0.8528029, *P*-value: 0.82904703 [TM-MSA versus TT-MSA]. (**F**) *q*-Value: −4.233, *P*-value: < 0.001 [WT, *n* = 13 versus MM-MSA, *n* = 9]; *q*-value: −0.265, *P*-value: 0.96192 [WT versus TT-MSA, *n* = 7]; *q*-value: 3.395, *P*-value: 0.00613 [MM-MSA versus TT-MSA].

First, we administered 10 µL of 50 mM fluorescent 2-deoxy-D-glucose to the nose of wild type (WT) mice to determine whether drugs could reach the brain. Fifteen minutes after injection, we removed the olfactory and confirmed the presence of fluorescent 2-deoxy-D-glucose. Immunofluorescence images showed clear signals in the glomerulus and the granular layer of the olfactory bulb (Fig. 1A). To further confirm that intranasally administered trehalose reached the brain, we investigated the kinetics of trehalose in the mouse whole brain using liquid chromatography-tandem mass spectrometry. Although trehalose was not detected as a peak in any of the groups (retention time 27.9 min), hexose was detected only in the brain of mice administered trehalose intranasally at a retention time of 16.4 min (Fig. 1B). Trehalose is a disaccharide composed of two glucose units linked by an α, α-1, 1-glycosidic linkage, which is degraded by trehalase localised in the brain^23^ and the brush border membrane of the small intestine and kidney in mammals.^24^ Since no hexose was detected in the brain of control and intravenous groups, intranasally administered trehalose may reach the brain, and be degraded to the hexose after catabolism.

### Trehalose improved memory impairment in the MSA model mice compared with maltose

We administered 10 µL of 2% trehalose or maltose intranasally to the MSA model mice once per week and gave water with 2% trehalose or maltose for two months after αSyn induction (Supplementary Fig. 2). We then performed the object-location memory task (Fig. 1C). Only (trehalose and trehalose) TT-MSA group (*n* = 6) displayed a higher exploration rate than the (maltose and maltose) MM-MSA group [(*n* = 6) (Fig. 1D)]. The exploration rate of the (maltose and trehalose) MT-MSA group (*n* = 4) and (trehalose and maltose) TM-MSA group (*n* = 4) did not significantly increase relative to MM-MSA group. Thus, we focused on the MM- and TT-MSA groups thereafter. To confirm the effect of trehalose on memory, we examined the long-term potentiation (LTP) in the hippocampus using TT-MSA group, MM-MSA group and WT mice. The LTP in the CA1 region was significantly impaired in the MM-MSA group mice (*n* = 9) compared with WT mice (*n* = 13). Notably, trehalose treatment reversed this impaired LTP [MM-MSA group (*n* = 9) versus TT-MSA group (*n* = 7), (Fig. 1E and F)]. These results suggest that trehalose attenuated the adverse effects of human αSyn that accumulated in the hippocampus.

### Trehalose increased αSyn phosphorylation in the cerebrum

**Figure 2:**
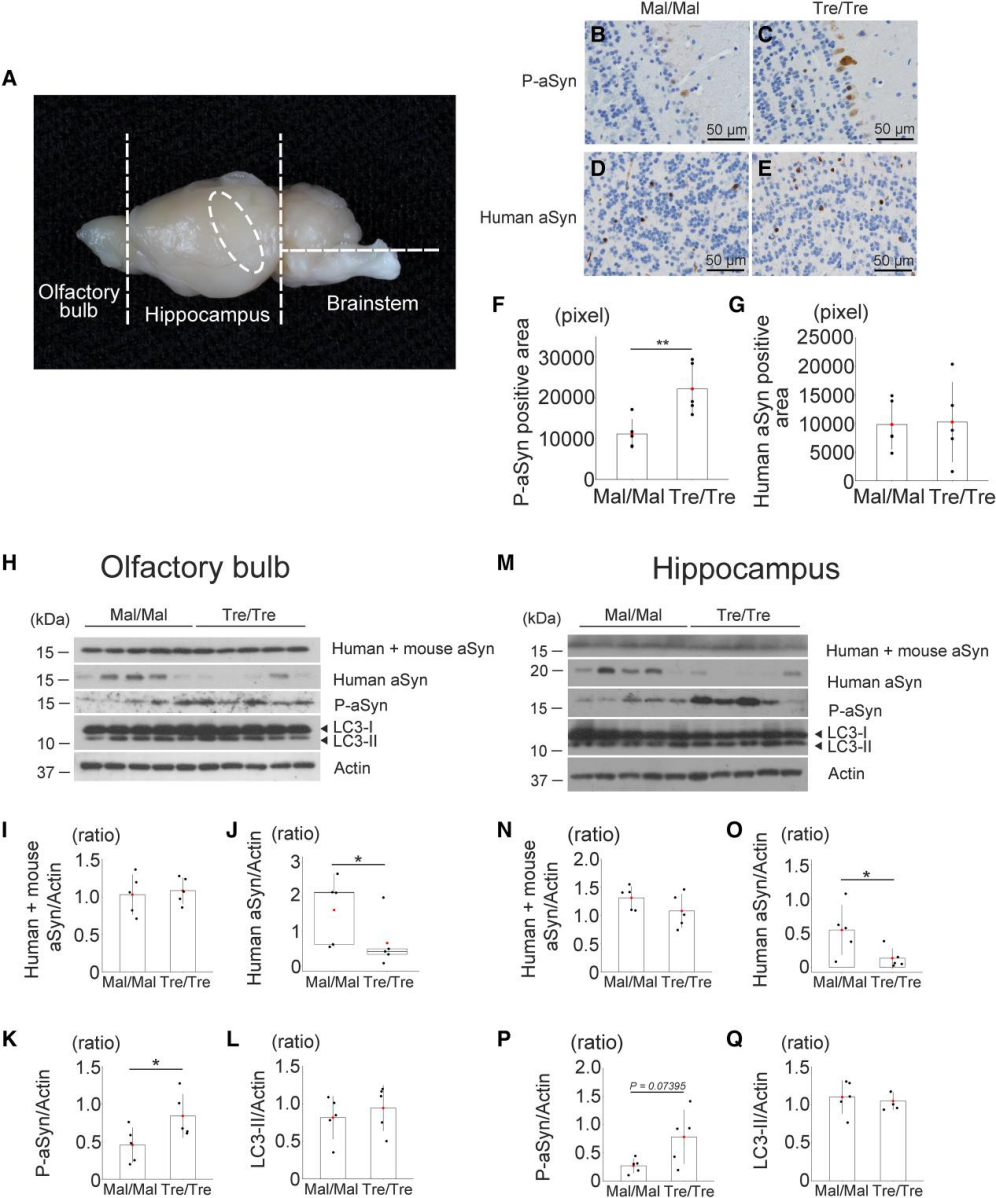
**Promotion of αSyn phosphorylation by trehalose.** (**A**) A schema shows the brain regions we used in the present study. (**B–E**) Immunoreactivity of phosphorylated αSyn (*P*-αSyn) and human αSyn in the olfactory bulb of the MSA model mice treated with maltose or trehalose. (**F**, **G**) Semi-quantification of P-αSyn and human αSyn positive area. **P < 0.01. (**H**) Western blot analysis of the olfactory bulb from MSA model mice treated with maltose or trehalose. (**I–L**) The expression levels of human + mouse αSyn, human αSyn, P-αSyn and LC3-II in the olfactory bulb. Intranasal and oral administration of trehalose (*n* = 5) significantly decreased human αSyn levels compared with maltose (*n* = 5). However, P-αSyn levels were significantly increased in trehalose-treated group compared with maltose group. *P < 0.05. (**M**) Western blot analysis of the hippocampus from MSA model mice treated with maltose or trehalose. (**N–Q**) The expression levels of human + mouse αSyn, human αSyn, P-αSyn and LC3-II in the hippocampus. Trehalose treatment (*n* = 5) significantly decreased human αSyn levels compared with maltose (*n* = 5), whereas P-αSyn levels tended to be increased in the trehalose treatment group (*P* = 0.07395). *P < 0.05. We used Mann–Whitney U-test for **J**, Welch two sample *t*-test for **P** and Student *t*-test for the other quantitative data in the figure. Test statistic: (**F**) *t*-value: −3.3949, *P*-value: 0.009431; (**G**) *t*-value: −0.11626, *P*-value: 0.9103; (**I**) *t*-value: −0.37286, *P*-value: 0.7189; (**J**) *U*-value: 23, *P*-value: 0.03175; (**K**) *t*-value: −2.3116, *P*-value: 0.04956; (**L**) *t*-value: −0.67834, *P*-value: 0.5167; (**N**) *t*-value: 1.4146, *P*-value: 0.1949; (**O**) *t*-value: 2.3422, *P*-value: 0.04725; (**P**) *t*-value: −2.3058, *P*-value: 0.07395; (**Q**) *t*-value: 0.47106, *P*-value: 0.6502. Uncropped blot data about panels **H** and **M** are shown in Supplementary material.

Next, to study how trehalose treatment can change human αSyn pathology, we examined MM- and TT-MSA groups (*n* = 5, each group) pathologically. Because we speculated that intranasally administered trehalose may reach the brain via the olfactory and/or trigeminal nerves,^18,19^ we sectioned the brains of MSA model mice as shown in Fig. 2A and then performed immunohistochemistry and western blotting analyses on the olfactory bulb, hippocampus and brainstem. Immunohistochemistry using the olfactory bulb showed that TT-MSA group had more phosphorylated αSyn (P-αSyn)-positive structures than MM-MSA group (Fig. 2B, C and F), whereas no difference was seen in human αSyn positive areas detected by syn211 antibody between the two groups (Fig. 2D, E and G). Trehalose and maltose treatment didn’t affect the area of oligodendrocytes (Supplementary Fig. 3). Immunoblot analysis also showed that the TT-MSA group had increased protein levels of P-αSyn compared with the MM-MSA group (Fig. 2H and K). Additionally, the TT-MSA group had lower levels of human αSyn detected by syn211 antibody than the MM-MSA group did (Fig. 2J). We then performed western blotting analysis using the hippocampus. The expression levels of human αSyn were significantly decreased and P-αSyn tended to be increased in the hippocampus of TT-MSA group compared with MM-MSA group (Fig. 2M, O and P). Human + mouse αSyn and LC3-II levels were unchanged between the two groups, however (Fig. 2I, L, N and Q). These findings suggested that trehalose administration promotes the αSyn phosphorylation in the cerebrum of the MSA model mice. No difference was found in the human and mouse *SNCA* mRNA levels (Supplementary Fig. 4).

### Trehalose reduced cytotoxic αSyn oligomers and accelerated αSyn aggregation

**Figure 3:**
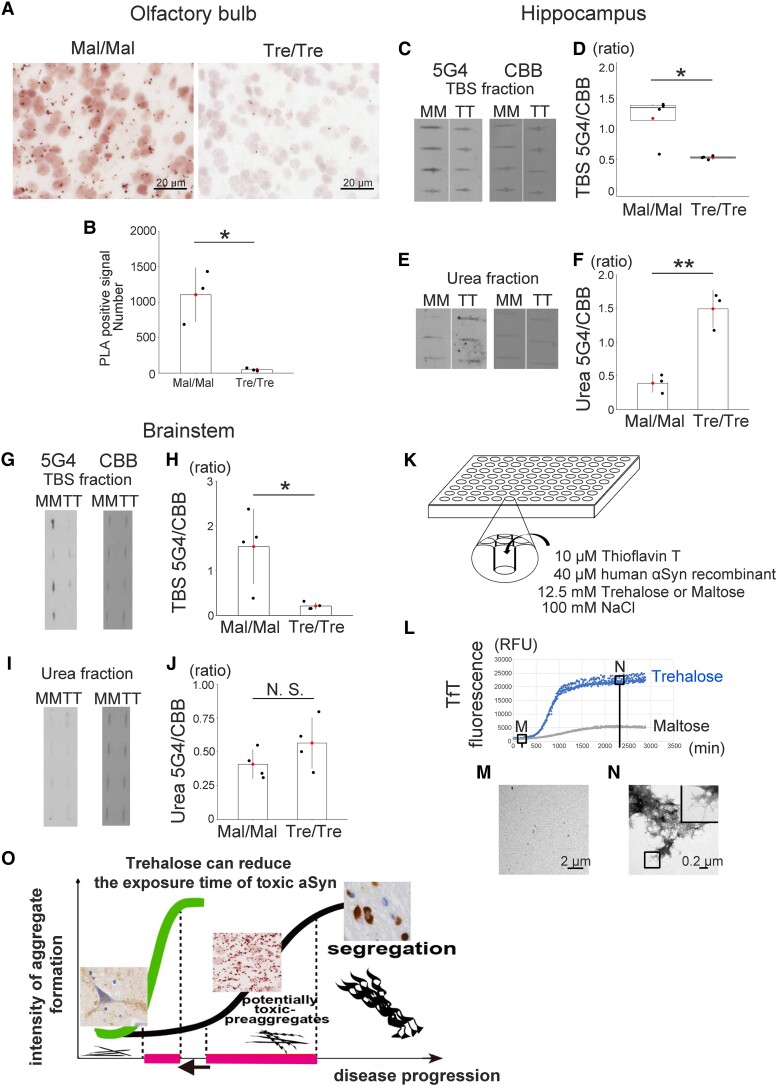
**Trehalose reduced the pre-aggregate form of αSyn by its unique property.** (**A**, **B**) Proximity ligation assay (PLA) with anti-Syn211 antibody showed a significant reduction of αSyn oligomers in the olfactory bulb of trehalose-treated mice (*n* = 3). (**C–F**) Filter trap assay using the hippocampus shows that trehalose treatment decreased the expression levels of αSyn oligomer in the TBS fraction (*n* = 4) and increased αSyn aggregation in the Urea fraction (*n* = 3) compared with maltose. Coomassie Brilliant Blue (CBB) staining indicates that almost equal amounts of sample were applied to samples from the MSA model mice. *P < 0.05, **P < 0.01. (**G–J**) Filter trap assay using the brainstem shows trehalose treatment (*n* = 4) reduced expression levels of αSyn oligomer compared with maltose treatment (*n* = 4) in the TBS-soluble fraction. CBB staining indicates that almost equal amounts of sample were applied to samples from the MSA model mice. *P < 0.05. (**K**) A schema of the in vitro kinetic analysis. We mixed the human αSyn recombinant protein with 100 mM NaCl, 10 µM thioflavin T and 12.5 mM trehalose or maltose, and mixture was reacted for 48 h at 37°C. (**L**) Trehalose accelerated αSyn aggregation, whereas maltose failed to aggregate αSyn. (**M**, **N**) We collected αSyn aggregates shortly after reaction started, and fully reacted αSyn aggregates. Transmission electron microscopy images show the formation of αSyn aggregates with increased incubation time. (**O**) Under pathological conditions, intermediate structures including oligomers, protofibrils and pre-aggregates were widely distributed throughout the brain (middle panel). These abnormal structures potentially exert cytotoxicity on neurons and glial cells, transforming into insoluble fibrils and aggregates and eventually forming neuronal and/or glial inclusions (right panel). To reduce cytotoxicity from intermediate aggregates (magenta lines), trehalose can help to wrap and separate them as quickly as possible, resulting in a shorter exposure time. The vertical axis indicates the degree of aggregation. We used Welch two sample *t*-test for **B** and **J**, Mann–Whitney U-test for **D** and Student *t*-test for **F** and **H**. Test statistic: (**B**) *t*-value: 4.7915, *P*-value: 0.04044; (**D**) *U*-value: 16, *P*-value: 0.02857; (**F**) *t*-value: −6.131, *P*-value: 0.003587; (**H**) *t*-value: 3.169, *P*-value: 0.04937; (**J**) *t*-value: −1.4368, *P*-value: 0.2008. Uncropped blot data about panels **C**, **E**, **G** and **I** are shown in Supplementary material.

Because soluble αSyn oligomers cause synaptic dysfunction and memory impairment in patients with MSA,^21,22^ we hypothesised that trehalose could reduce the levels of cytotoxic αSyn oligomers by accelerating the formation of αSyn aggregates. Therefore, we performed PLA or filter trap assay, which detects αSyn oligomers.^21,25,26^ PLA showed that TT-MSA group (*n* = 3) had lower positive signals compared with MM-MSA group in the olfactory bulb (*n* = 3) (Fig. 3A and B). Next, we performed filter trap assay using 5G4 antibody, which reacts with aggregated αSyn including oligomers and fibrils on this assay.^27^ Trehalose treatment reduced soluble αSyn oligomer in the TBS fraction of the hippocampus (Fig. 3C and D). Importantly, TT-MSA group had higher levels of αSyn aggregates in the insoluble fraction (Urea fraction) than MM-MSA group (Fig. 3E and F). In addition, we performed PLA and filter trap assay using the brainstem. Despite no difference in αSyn aggregates in the Urea fraction between the two groups (Fig. 3I and J), trehalose treatment significantly reduced αSyn oligomers in the brainstem (Fig. 3G and H, Supplementary Fig. 5). Taken together, these results suggested that trehalose treatment reduced αSyn oligomers and accelerated αSyn aggregation.

### Trehalose exerted an aggregating effect on αSyn in nature

We previously reported that trehalose strongly activated autophagy flux, which reduced αSyn-synphilin-1 aggregates in a cultured cell model.^17^ Consistent with the findings of our previous report,^17^ HeLa cells treated with trehalose showed significantly increased protein levels of LC3-II compared with those treated with maltose (Supplementary Fig. 6). However, such autophagy activation was not found in the mouse brain of trehalose-treated MSA mouse model (Fig. 2L and Q). Therefore, to study whether trehalose accelerates aggregate formation, we performed *in vitro* kinetic analyses using thioflavin T. We reacted 40 µM human αSyn recombinant protein with 100 mM NaCl, 12.5 mM trehalose or maltose, and 10 µM thioflavin T for 48 h at 37°C (Fig. 3K). The fluorescence intensity changed more rapidly with 12.5 mM trehalose treatment than with maltose treatment (Fig. 3L). We further performed transmission electron microscopy. Transmission electron microscopy images confirmed the formation of αSyn aggregates along with increased incubation time (Fig. 3M and N). These findings support an effect of trehalose in accelerating the formation of αSyn aggregates.

## Discussion

αSyn species do not exist in isolation in synucleinopathies, but rather as a monomeric form of αSyn, which, under pathological conditions, changes into an oligomeric form and then into fibrils in a continuum. The more cytotoxic αSyn species neurones are exposed to, the more likely they are to lose neuronal function. Several lines of evidence have revealed that in *in vitro* or *in vivo* models of Lewy body disease αSyn oligomers impair synaptic functions and suppress LTP, which reflects the activity of memory storage processes.^28-31^ In the present study, we demonstrated that trehalose reduced the amount of cytotoxic oligomer and increased the aggregated form of αSyn in the early stages of disease. Accordingly, memory was restored in the trehalose-treated MSA mouse model. Previous reports have suggested that the formation of αSyn aggregates may represent a cellular survival process.^4-6^ Although we could not capture the transitional state of oligomers to aggregates directly, our results indicate that trehalose improved memory by converting cytotoxic αSyn oligomers into aggregates (Fig. 3O).

We have also shown for the first time that the cytoprotective effects of trehalose can be enhanced by a combination of oral and intranasal administration of trehalose. Sarkar *et al*.^32^ showed that oral administration of trehalose rescued motor deficits by reducing the 1-methyl-4-phenyl-1,2,3,6-tetrahydropyridine-induced loss of tyrosine hydroxylase and dopamine transporter in mice. Additionally, trehalose has been found to reduce the levels of potentially toxic proteins.^17,33,34^ In these reports, the oral administration of trehalose improved the clinical phenotype. Consistent with previous reports, trehalose treatment improved memory function in MSA model mice, whereas trehalose reduced αSyn oligomers by accelerating aggregate formation. Our *in vitro* kinetic assay supported the hypothesis that trehalose directly accelerates αSyn aggregate formation. Trehalose exerts chemical chaperone activity,^34,35^ which may be responsible for the formation of αSyn aggregates. Although trehalose is also reported to inhibit β-amyloid aggregates and may play a paradoxical role among neurodegenerative diseases,^36,37^ these findings suggest that trehalose accelerates αSyn aggregate formation by its chaperone activity.

In the present study, trehalose treatment improved memory in the MSA mouse model by reducing αSyn oligomers, while this treatment increased αSyn phosphorylation at serine-129. Our previous study showed that P-αSyn at serine-129 was detected in the high-density fraction of a sucrose density gradient using tissues from MSA patients.^20^ Consistent with our previous findings, the present study showed increased protein levels of phosphorylated and aggregated forms of αSyn in the hippocampus (Figs. 2P and 3E and F). However, the role of αSyn phosphorylation still remains to be elucidated. Fujiwara *et al*.^38^ reported that αSyn phosphorylation at serine-129 was associated with aggregate formation. By contrast, another study suggested an inhibitory effect on disease modification.^39^ Surprisingly, others found no effect of αSyn phosphorylation on disease modification.^40,41^ Furthermore, αSyn can be phosphorylated even under physiological conditions^42^ and physiological P-αSyn is reported as a synaptic transmission regulator.^43^ It would be interesting to investigate how and when αSyn phosphorylation affects neurodegeneration.

In the present study, we administered trehalose orally and/or intranasally to our MSA model mice. In the oral route, trehalose is degraded into two glucoses, absorbed from the small intestine into the blood and eventually crosses the blood–brain barrier to reach the brain. In contrast, intranasal administration of trehalose can bypass the blood–brain barrier via the olfactory and/or trigeminal nerves^18,19^ and reach the brain directly without being degraded in the digestive tract. This route may provide high bioavailability and a shorter time to have an effect.^44^ There are potential limitations to trehalose treatment in different species, as in rodents, 50% of the surface area of the nasal cavity is covered by the olfactory epithelium, compared with 8% in humans.^44^ Although these coverage differences may affect the efficacy of intranasal administration of trehalose in humans, and dosage might need further optimization, our results suggest that this is a promising therapeutic approach.

## Conclusion

We propose a novel strategy for the treatment of MSA, in which the acceleration of aggregate formation leads to less exposure to toxic αSyn oligomers, particularly in the early phase of disease progression.

## Supplementary Material

fcae193_Supplementary_Data

## Data Availability

All data generated or analysed during this study are included in this published article and its supplementary information files.
