# Supplementary material for: Intranasal administration of trehalose reduces α-synuclein oligomers and accelerates α-synuclein aggregation
Source: Brain Commun. 2024 Aug 20;6(4):fcae193. doi: 10.1093/braincomms/fcae193 (PMC11334933; doi:10.1093/braincomms/fcae193)
Supplement: fcae193_Supplementary_Data [file fcae193_supplementary_data.pdf]

Supplementary material

Supplementary method

Behavioural analysis

To assess the memory of the multiple system atrophy (MSA) model mice, they were subjected to an object-location memory task test[1, 2] consisting of three parts: familiarisation, an acquisition trial, and a test trial. During these three parts, the time spent exploring each object was recorded using a video tracking system (CaptureStar, CleverSys Inc., Reston, VA, USA). Briefly, the mice were placed in a box for 1 h for habituation. Next day, they were placed in the same box containing two objects (O1 and O2) located in the upper-left (O1) and upper-right (O2) corners. The mice explored O1 and O2 for 5 min (acquisition period). After the acquisition period, O2 was moved to the lower right corner (O2 [LR]). The mice explored O1 and O2 (LR) for 5 min (test period). Mice normally have a spontaneous tendency to spend more time exploring a 'new' object [O2 (LR)] than the original object. The exploration rate (%) was calculated as follows: [mouse interaction time (s) with O2 (LR) / mouse interaction time with O1 + O2 (LR)] × 100. A high exploration rate indicated good memory function. The exploration of an object was defined as positioning the nose towards an object at a distance of < 1 cm and/or touching the object with the nose. Turning around or sitting on an object was not considered exploration. The mouse behaviour was analysed from the recorded images by trained observers blinded to the treatment conditions.

Measurement of trehalose by liquid chromatography-tandem mass spectrometry (LC-MS/MS)

2% sodium dodecyl sulphate (SDS)/8M urea was added to the excised mouse whole brain at 0.1% w/v. The brain was homogenised by sonication and centrifuged at 15,000 g and 4°C for 10 min. The supernatant was assayed for protein by bicinchoninic acid assay and the concentration was adjusted to 12 mg/ml. 0.2 ml of the lysate was mixed with 1 ml of 80% acetonitrile containing 2 mM ammonium acetate and centrifuged at 15,000g and 4°C

for 10 min, and the resulting supernatant was subjected to LC-MS/MS, a high-performance liquid chromatography (HPLC) system (ExionLC AD, AB SCIEX) coupled to a QTRAP6500+ mass spectrometer (AB Sciex) in electrospray ionization mode. Trehalose was analysed in negative mode. Ten microlitres of the samples were injected into a high-performance carbohydrate column (250 mm length, 4.6 mm inner diameter, 4 μm particle, Waters) at 35 °C and eluted with an isocratic elution of 80% acetonitrile containing 2 mM ammonium acetate. Mass spectrometry settings are as follows: curtain gas, 30; ion spray voltage, -4500 V; temperature, 300°C; ion source gas 1, 50 psi; ion source gas 2, 60 psi; CAD gas, 7 psi; input potential, -10 V. The flow rate was set at 1.0 ml/min. Trehalose was identified and quantified using an external standard and the optimised multiple reaction monitoring setting of 340.9 m/z and 178.9 m/z for the precursor ion (Q1) and product ion (Q3), respectively. The declustering potential (DP), collision energy (CE) and collision cell exit potential (CXP) were set to -160 V, -18 V and -17 V respectively. For hexose measurements Q1, Q3, DP, EP and CXP were set to 178.8 m/z, 88.9 m/z, -85 V, -10 V and -11 V respectively.

Electrophysiological analysis

The mice were deeply anaesthetised with three mixed anaesthetics (0.75 mg/kg medetomidine, 4.0 mg/kg midazolam, and 5.0 mg/kg butorphanol), and the brains were rapidly removed and placed in oxygenated cold-cut artificial cerebrospinal fluid (ACSF) containing 92 mM NaCl, 2.5 mM KCl, 1.2 mM NaH_2PO_4, 10 mM $MgSO_4$, 2.0 mM $CaCl_2$, 30.0 mM $NaHCO_3$, 5.0 mM sodium ascorbate, 2.0 mM thiourea, 3.0 mM sodium pyruvate, 20.0 mM 4-(2-hydroxyethyl)-1-piperazineethanesulfonic acid (HEPES), and 25.0 mM glucose. Transverse hippocampal slices (350-μm thick) were prepared using a micro-slicer (Vibratome3,000, Vibratome, MO, USA). The prepared hippocampal slices were incubated at 32°C for 12 min in choline chloride-based recovery ACSF containing 92 mM NaCl, 2.5 mM KCl, 1.2 mM NaH_2PO_4, 10 mM $MgSO_4$, 2.0 mM $CaCl_2$, 30.0 mM $NaHCO_3$, 5.0 mM sodium ascorbate, 2.0 mM thiourea, 3.0 mM sodium pyruvate, 20.0 mM HEPES, and 25.0 mM glucose. After the recovery incubation, the slices were kept for at least 1 h at room temperature (25°C) in recording ACSF containing 126 mM NaCl, 2.5 mM KCl, 1.25 mM NaH_2PO_4, 1.2 mM $MgSO_4$, 2.0 mM $CaCl_2$, 26.0 mM $NaHCO_3$,

and 20.0 mM glucose. All the solutions used in this experiment were saturated with 95% O_2 and 5% CO_2 prior to use. The chamber of the recording system (MED probe; Alpha MED Scientific, Osaka, Japan) was perfused with ACSF (2 ml/min at 30°C). Field-evoked potentials (fEPSPs) in the CA3 region were recorded using a multielectrode array system (MED64, Alpha MED Scientific) for 100 ms at a sampling rate of 10 kHz. Stimulation for fEPSPs was delivered at a rate of 1 stimulus every 20 s. The stimulation intensity was sufficient to evoke fEPSPs that were 30% of the maximum fEPSP slope (10–90%). To induce LTP, theta burst stimulation was applied 15 min after the start of recording. To assess LTP, Mobius software (Alpha MED Scientific) was used for offline data analysis of the fEPSP slope. The magnitude of LTP was measured as a percentage of the baseline fEPSP slope during the 15 min immediately before theta burst stimulation. For statistical analysis of the magnitude of LTP in the three groups, the average fEPSP slope of the last 15 min was compared using one-way ANOVA.

Pathological analysis

The mice were anaesthetised with isoflurane (1119, Pfizer, New York, NY, USA). Mice were transcardially perfused with phosphate buffer. Then, the brain was removed and the right hemisphere was fixed with 4% paraformaldehyde for 48 h. After dehydration using a graded ethanol series, the right hemisphere was embedded in paraffin and cut into 4-μm-thick sections.

For immunohistochemistry, the sections were dehydrated and pre-treated with heat retrieval using an autoclave for 10 min in 10 mM citrate buffer (pH 6.0). The sections were then subjected to immunohistochemical processing using the avidin-biotin-peroxidase complex method (PK-6100; Vector Laboratories, Burlingame, CA) with diaminobenzidine (Sigma-Aldrich, St Louis, MO) and counterstained with haematoxylin.

Immunoblot analysis

The brain samples collected from the left hemisphere were weighted and homogenised with 20 volumes of buffer containing 4% SDS (75 mM Tris-HCl, pH 6.8), 25% glycerol,

and 5% β-mercaptoethanol. The samples were passed through a 23-gauge needle attached to a 1-ml syringe and centrifuged at 12,000 rpm for 3 min.

The samples were resolved on an SDS-PAGE gel: 5-20 % Tris-glycine gradient gel (HER-R520L, ATTO, Tokyo, Japan) using an electrophoresis system (AE-6530M, ATTO) under constant current (50 mA for 1 h) and then transferred to a methanol-activated polyvinylidene difluoride membrane (IPVH00010, EMD Millipore, Burlington, MA) using Mini Trans-Blot cell system (1703930JA, Bio-Rad, Hercules, CA) under constant voltage (90 V, 1 h) at 4 °C. For the detection of α-synuclein and phosphorylated α-synuclein, the membranes were immersed in 0.4 % paraformaldehyde in 0.01 M phosphate-buffered saline overnight at 4 °C before membranes were blocked. The membranes were blocked for 1 h at 25 °C with blocking buffer [20 mM Tris-HCl, pH 7.5, 133 mM NaCl and 0.1 % Tween 20 (TBS-T) containing 5 % skimmed milk]. The membranes were then washed with washing buffer (TBS-T containing 1 % skimmed milk). After washing, the membranes were incubated with primary antibodies (Supplementary Table 2) for 1 h at 25 °C. The membranes were washed with TBS-T and incubated with HRP-conjugated secondary antibodies (Supplementary Table 2) for 1 h at 25 °C. Signal detection was performed according to the protocol provided by enhanced chemiluminescence (ECL) or ECL prime detection system (RPN2232; RPN2209; GE Healthcare Bioscience, Marlborough, MA, USA). Positive signals were semi-quantified using ImageJ software (NIH). Actin was used for normalisation.

Quantitative reverse transcription-polymerase chain reaction

Total RNA was extracted from the right hemisphere brain of the maltose/maltose or trehalose/trehalose group (N = 3 per group) using an RNeasy Lipid Tissue Mini Kit (QIAGEN, Hilden, Germany). Moreover, cDNA was synthesised from 5 μg of total RNA using SuperScript^{TM} III Reverse Transcriptase (Invitrogen). An aliquot of cDNA was used for gene expression analysis using the SsoFast^{TM} EvaGreen® Supermix (Bio-Rad; Hercules, CA, USA) and CFX Real-Time PCR Detection System (Bio-Rad) with the following primer sets: human *SNCA* (5’-GCATGGTGTGGCAACAGT-3’; 5’-TGTCACACCCGTCACCAC-3’) and mouse *SNCA* (5’-

TGACGGGTGTGACAGCAGTAG-3'; 5'-CAGTGGCTGCTGCAATG-3'). Mouse *Gapdh* (5'-AGAACATCATCCCTGCATCCA-3'; 5'-CCGTTCAGCTCTGGGATGAC-3') was used for normalisation.

Sample preparation for filter trap assay

Frozen brainstem and hippocampus from MSA model mice were used for filter trap assay. These samples were aliquoted and frozen at -80°C until used for filter trap assay.

Brainstem

First, the brainstems were weighted and homogenised with 10 volumes of tris-based buffer (30 mM Tris-HCl (pH 7.5), 150 mM NaCl, 10% glycerol). After homogenization, the samples were passed through a 23-gauge needle and centrifuged at 1,000 x g for 10 min at 4°C [tris-buffered saline (TBS) fraction]. Subsequently, the pellet was weighted and two volumes of 2 % triton buffer (30 mM Tris-HCl (pH 7.5), 150 mM NaCl, 10% glycerol, 2 % Triton X-100) were added. The samples were passed through a 23-gauge needle and incubated for 30 min at 37 °C with 70 rpm shaking. After incubation, the samples were centrifuged at 14,500 x g for 30 min at 4°C (Triton fraction). Finally, the resultant pellet was sonicated in a volume of 8 M Urea (Urea fraction).

Hippocampus

We collected the TBS fraction as same method for the brainstem. The pellet was weighted and a volume of 2 % triton buffer were added. The samples were passed through a 23-gauge needle and incubated for 30 min at 37 °C with 70 rpm shaking. After incubation, the samples were centrifuged at 14,500 x g for 30 min at 4 °C (Triton fraction). Subsequently, the pellet was weighted and a volume of 2 % of sarkosyl buffer (30 mM Tris-HCl (pH 7.5), 150 mM NaCl, 10% glycerol, 2 % N-lauroyl-sarcosine). The samples were passed through a 23-gauge needle and incubated 30 min at 37 °C. After incubation, the samples were centrifuged at 14,500 x g for 30 min at 4 °C (Sarkosyl fraction). Finally, the resultant pellet was sonicated in two volume of 8 M urea (Urea fraction).

Filter trap assay

To detect native Syn, we modified the previously described method of filter trap analysis[3]. Briefly, fractionated samples were applied to a 0.22-μm cellulose acetate membrane (Advantec, Tokyo, Japan) on a slot blot apparatus (Bio-Rad) using a vacuum manifold. We washed the membrane with TBS containing 0.1% Tween20, then the membrane was blocked for 1 hour at 25°C using blocking buffer. The subsequent steps were the same as for Western blotting analysis. After signal detection, membranes were stained with Coomassie Brilliant Blue for 10 minutes, and then washed with 10% acetic acid until the background was de-stained. Positive signals were evaluated semi-quantitatively using ImageJ software (NIH) and Coomassie Brilliant Blue-stained bands were used for normalisation.

Proximity ligation assay

Proximity ligation assay (PLA) was performed as previously described, with minor modifications[2, 4], using Duolink PLA (DUO92009; DUO92010; DUO92012; Merck Millipore). The conjugates were prepared using Duolink Probemaker Plus and Minus kits with the bovine serum albumin and azide free α-synuclein (Syn211) antibody (ab206675; Abcam, Cambridge, UK). Paraffin-embedded tissues were dehydrated and pre-treated by heat retrieval in an autoclave for 10 min in 10 mM citrate buffer (pH 6.0). The sections were treated with Duolink block solution at 37°C for 1 h, and then with α-synuclein (Syn211) antibody conjugates diluted in Duolink PLA diluent (1:100) overnight at 4°C. Then, the sections were washed with Tris-based buffer containing 0.05% Tween-20 and incubated with Duolink ligation reagents for 1 h at 37°C, followed by Duolink amplification reagents for 2.5 h at 37°C. Finally, the sections were washed and incubated with Duolink detection solution for 1 h at room temperature (25°C) and counterstained with haematoxylin.

Cultured cells and treatment

HeLa cells (Japanese Collection of Research Bioresources Cell Bank, Osaka, Japan) were maintained in Dulbecco's modified Eagle's medium supplemented with 10% fetal bovine serum and 1 % antibiotic and incubated at 37 °C under 5 % CO_2. We seeded HeLa cells (cell density: 1.0 x 10^5 cells/dish) on 35-mm dishes and added 2% of maltose or trehalose.

After 24 hours, treated cells were collected with 100 µL of buffer containing 4% SDS (75 mM Tris-HCl, pH 6.8), 25% glycerol, and 5% β-mercaptoethanol. The samples were passed through a 23-gauge needle attached to a 1-ml syringe and centrifuged at 12,000 rpm for 3 min.

In vitro kinetic assay

An *in vitro* kinetic assay was performed to detect α-synuclein aggregates according to a previously described protocol[5]. α-Synuclein polymerisation reactions were performed using 40 µM of α-synuclein recombinant protein (Rpeptide, GA; Abclonal, Tokyo, Japan) with 100 mM NaCl and 12.5 mM disaccharide at 37°C and monitored by the fluorescence of 10 µM Thioflavin T (ThT). The samples were agitated every 10 seconds. ThT emissions were measured every 4 min for 48 h at λmax = 477 nm upon excitation at 450 nm. To increase the reproducibility of data between wells, we used one Teflon ball (17649-100, Polysciences, Warrington, PA, USA) per well. The plates were sealed with clear polyolefin sealing tape (Thermo Scientific, Waltham, MA, USA) and measured using FlexStation3 (Molecular Devices, Sunnyvale, CA, USA). The photomultiplier gain was set to low (i.e., six flashes per reading), and the signals were read from the bottom of the plate.

Transmission electron microscope

Recombinant αSyn (40 µL) was incubated in Tris-based buffer containing NaCl and ThT at 37°C. After glutaraldehyde (final concentration = 2.5%) was added to the samples, they were centrifuged at 15,000 × g for 20 min at 4°C. The samples (5 µL) from different time points were applied to film-coated and carbon-coated copper grids (Nissin EM, Tokyo, Japan) and incubated for 5 min. Excess samples were removed from the edge of the grid using a filter paper. Then, the grids were washed three times with filtered distilled water and blotted onto a filter paper. Negative staining was performed with 2% filtered uranyl acetate for 5 min. Excess dye was blotted onto a filter paper, washed three times with filtered distilled water, and air-dried for 10 min. Photomicrographs were obtained using a JEOL 1230 electron microscope (JEOL Ltd., Tokyo, Japan) at an accelerating voltage of 80 kV.

Quantitative analysis

Phosphorylated αSyn and human αSyn positive areas were semi-quantified in 5 fields of the olfactory bulb from MSA model mice (N = 5) at x20 original magnification. Olig2 positive areas were semi-quantified using the dentate hilus of MSA model mice (N = 5). Semi-quantitative analyses were performed by the Image J software (NIH, Bethesda, MD). Positive signals were isolated by the colour deconvolution plugin in ImageJ. The images were then converted to binary image and quantified positive signal areas.

The number of PLA signals was counted in 4 fields of the olfactory bulb and brainstem from MSA model mice (N = 3) at x40 original magnificent and the average number of these signals was then calculated.

Supplementary Reference

1. Murai, T., Okuda, S., Tanaka, T., Ohta, H. (2007) Characteristics of object location memory in mice: Behavioral and pharmacological studies. *Physiol. Behav*. 90, 116-124.
2. Miki, Y., Tanji, K., Shinnai, K., Tanaka, M.T., Altay, F., Foti, S.C., et al. (2022) Pathological substrate of memory impairment in multiple system atrophy. *Neuropathol. Appl. Neurobiol.* 487, e12844.
3. Tanji, K., Tanaka, T., Mori, F., Kito, K., Takahashi, H., Wakabayashi, K., et al. (2006) NUB1 suppresses the formation of Lewy body-like inclusions by proteasomal degradation of synphilin-1. *Am. J. Pathol.* 169, 553-565.
4. Roberts, R.F., Wade-Martins, R., Alegre-Abarrategui, J. (2015) Direct visualization of alpha-synuclein oligomers reveals previously undetected pathology in Parkinson's disease brain. *Brain* 138, 1642-1657.
5. Wordehoff, M.M., Hoyer, W. (2018) alpha-Synuclein Aggregation Monitored by Thioflavin T Fluorescence Assay. *Bio Protoc.* 8, e2941.

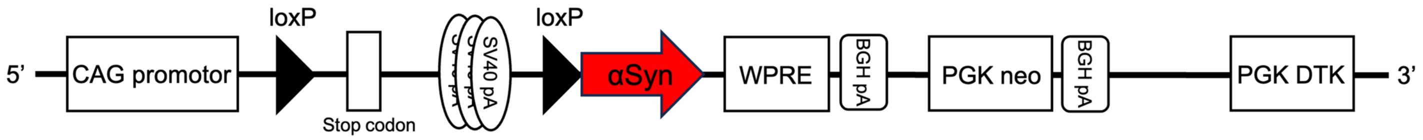

Supplementary Figure 1

A schema of the inserted gene into the Rosa 26 locus. When Cre recombinase are inactive, human αSyn expression is prevented by the stop codon located between the two loxP sequences. After injection of tamoxifen, Cre recombinase splices out the sequence between the two loxP sequences and MSA model mice start to express human αSyn in oligodendrocytes.

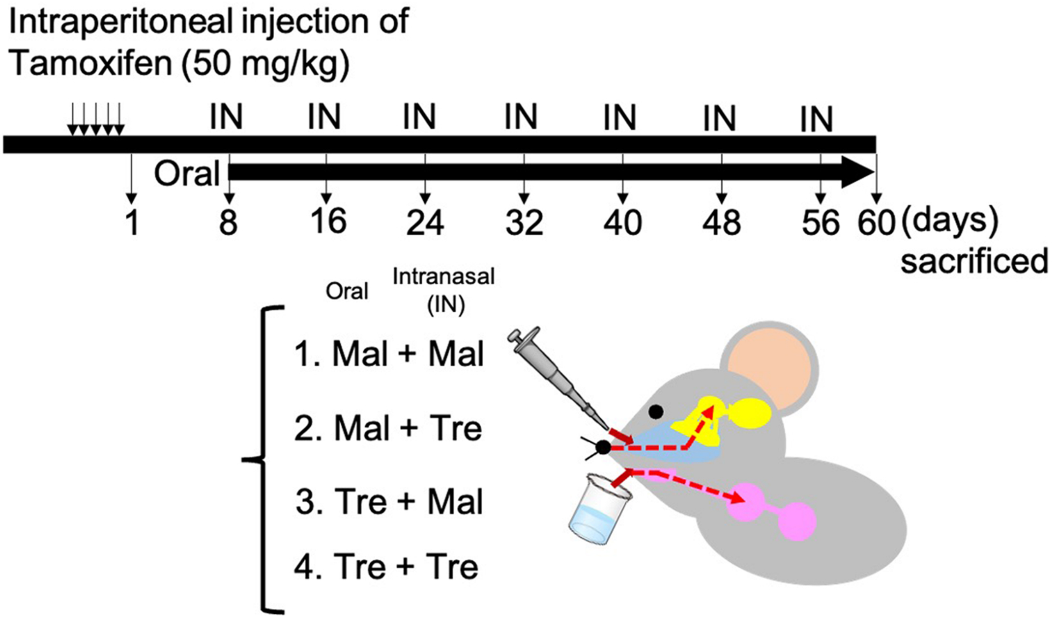

Supplementary Figure 2

Schema of the time course of the experimental design. After intraperitoneal tamoxifen injection for five days, we investigated the effect of trehalose/maltose on MSA model mice. Four groups consisting of combinations of trehalose and maltose based on intranasal and oral administration patterns were used.

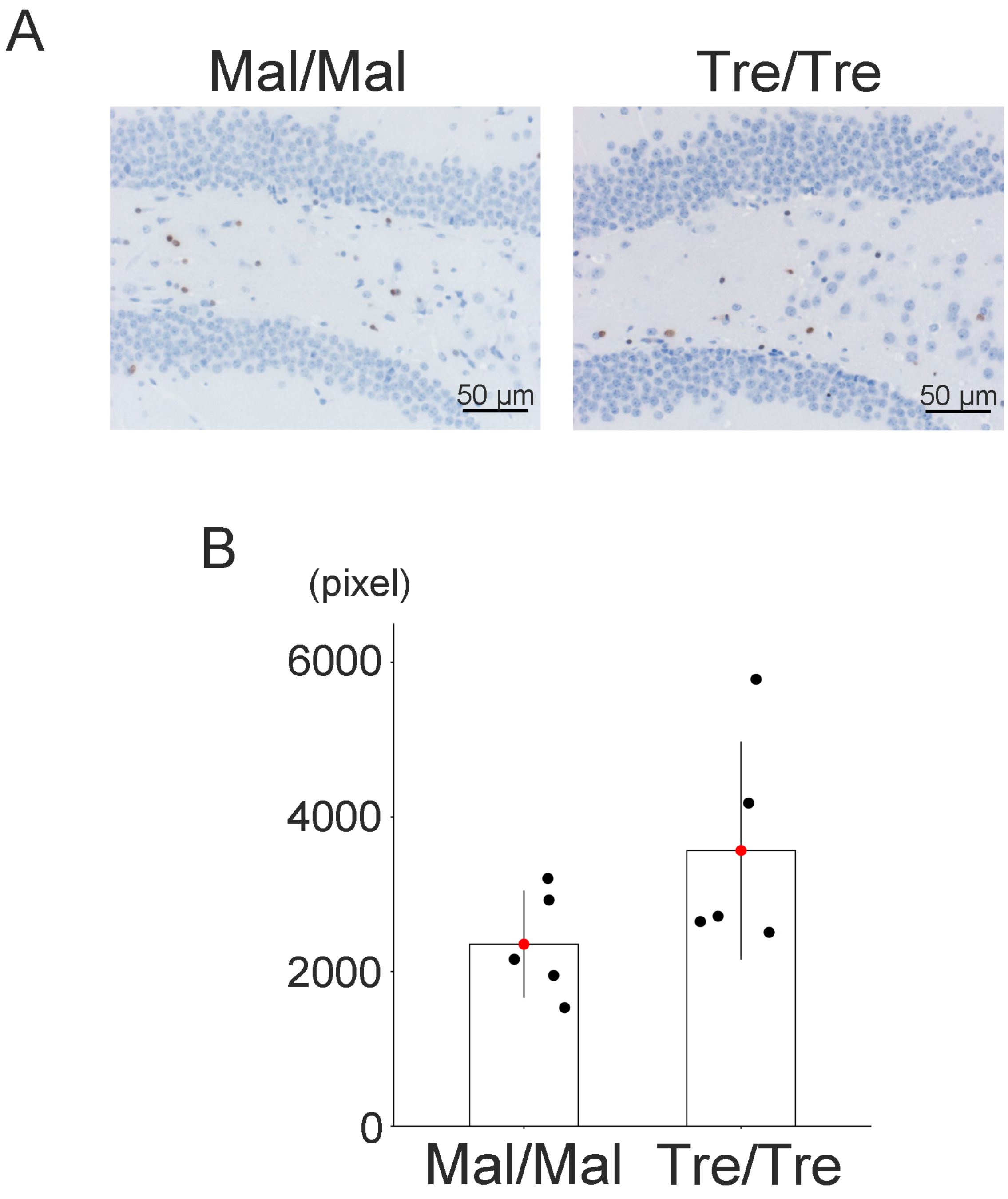

Supplementary Figure 3

Immunohistochemistry using anti-Olig2 antibody (A, B) showed no differences in Olig2 positive signals in the dentate hilus between the MM- and TT- MSA groups. We used Student t-test for (B). Test statistics: t value: -1.723, p value: 0.1232.

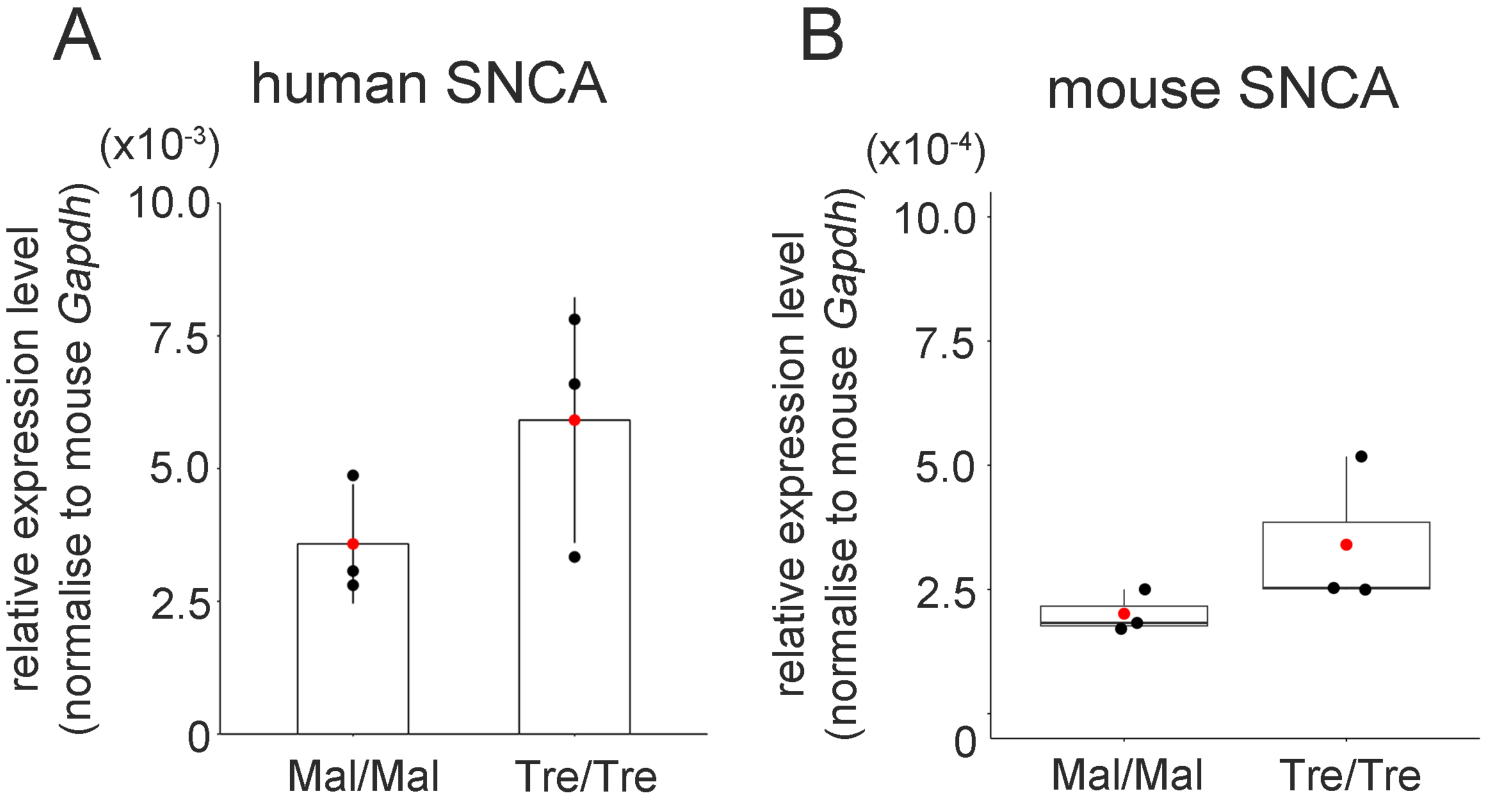

Supplementary Figure 4

Total RNA was extracted from the right hemisphere of MSA model mice treated with maltose (N = 3) or trehalose (N = 3) 2 months after αSyn induction. (A, B) Reverse transcription and qRT-PCR analysis shows no significant differences in human and mouse *SNCA* mRNA levels. We used Student t-test for (A) and Mann-Whitney U-test for (B). Test statistics: (A) t value: -1.5694, p value: 0.1916; (B) U value: 1, p value: 0.2.

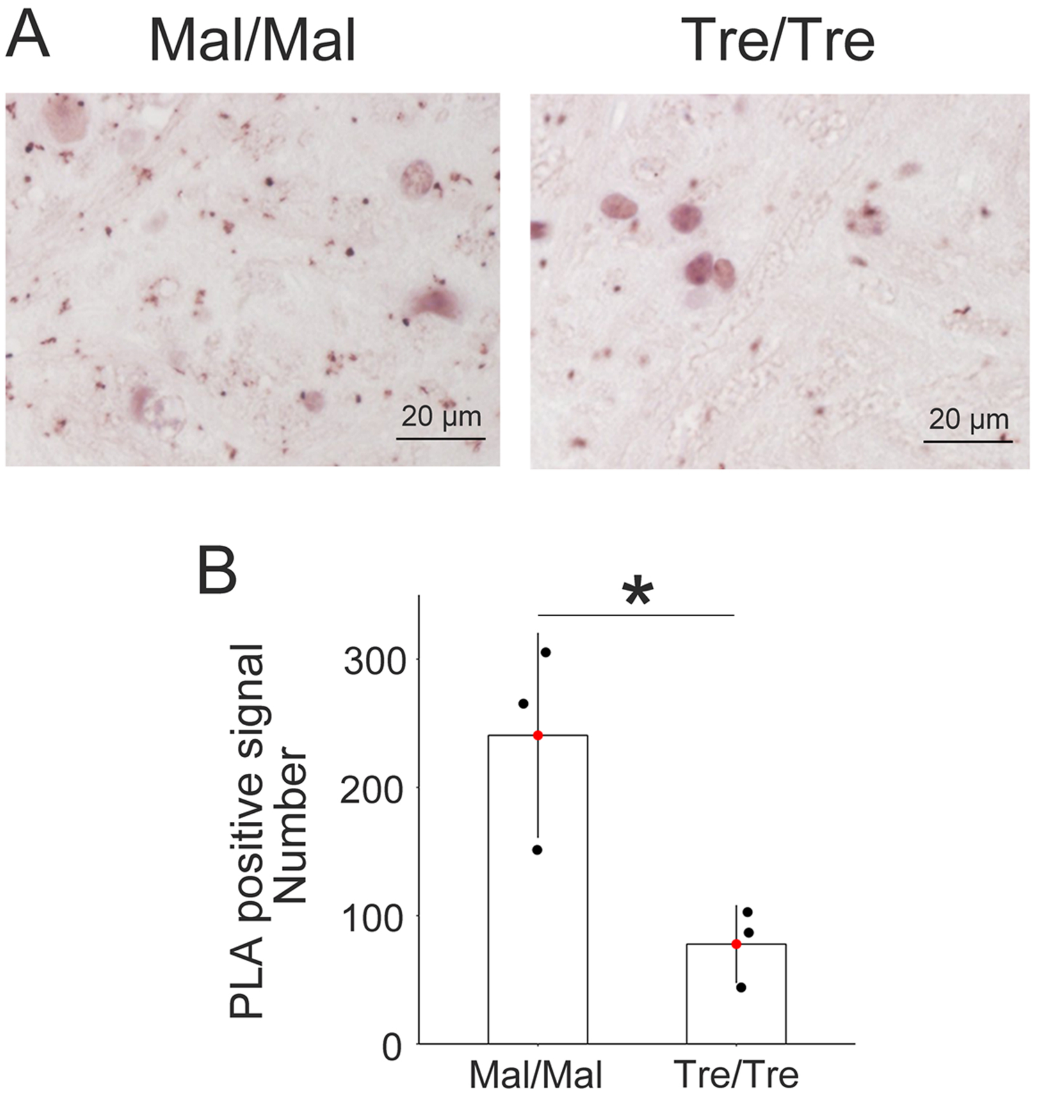

Supplementary Figure 5

Proximity ligation assay (PLA) using anti-Syn211 antibody shows significant reduction of αSyn oligomers in the brainstem from trehalose-treated mice (N = 3). $*P < 0.05$. We used Student t-test for (B). Test statistic: (B) t value: 3.2975, p value: 0.03.

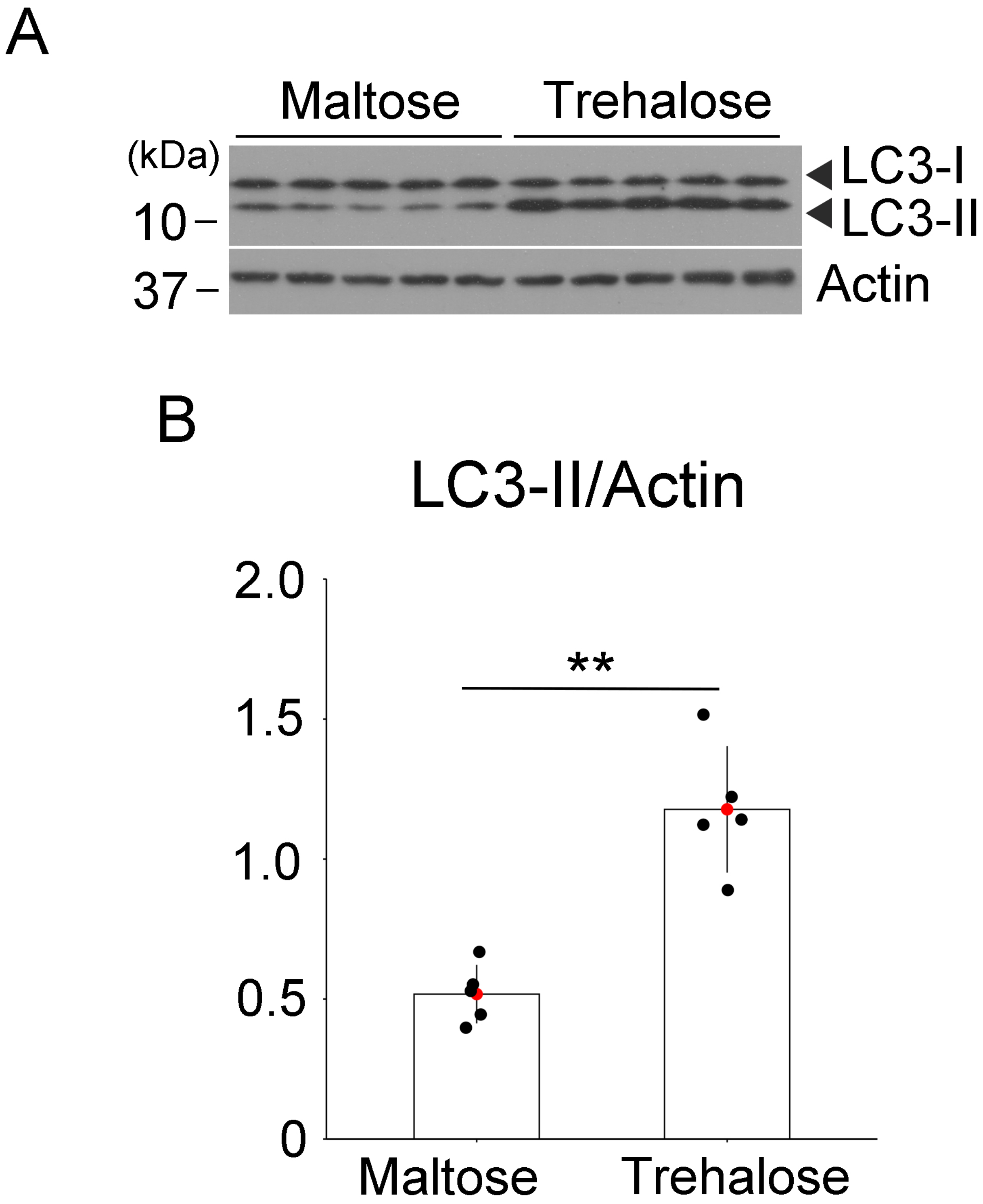

Supplementary Figure 6

HeLa cells were treated with 2% maltose or trehalose for 24 hours. (A, B) HeLa cells treated with trehalose showed significantly increased protein levels of LC3-II compared with those treated with maltose. $**P < 0.01$. We used Student's t test for (B). Test statistics: t value: -5.9293, p value: 0.00035.

Supplementary Table 1: Demographic data of mice in the present study.

Group	Treatment	Number (total number = 71)	Age (week) (average, 32.15 weeks)	Gender (male: female)
**Liquid chromatography-tandem mass spectrometry (Fig. 1B)**				
Control	Non	1	61	0 : 1
Control	Intravenous injection of trehalose	1	61	0 : 1
Control	Nasal administration of trehalose	1	61	0 : 1
**Behavioural analysis (Fig.1D)**				
MSA model mouse	Maltose/maltose	6	25.67	1 : 2
MSA model mouse	Trehalose/maltose	4	30.25	1 : 3
MSA model mouse	Maltose/trehalose	4	27.75	1 : 3
MSA model mouse	Trehalose/trehalose	6	28.17	1 : 1
**Electrophysiological analysis (Fig. 1E, F)**				
Control	Non	3	26	1 : 0
MSA model mouse	Maltose/maltose	2	25.5	1 : 0
MSA model mouse	Trehalose/trehalose	2	26.5	1 : 0
Numbers in the manuscript indicate the number of hippocampal neurons examined.				
**Immunohistochemistry (Fig. 2B-G, Supplementary Fig. 3)**				
MSA model mouse	Maltose/maltose	5	36	2 : 3
MSA model mouse	Trehalose/maltose	5	36	2 : 3
**Immunoblotting (Fig. 2H-Q)**				
MSA model mouse	Maltose/Maltose	5	36	2 : 3
MSA model mouse	Trehalose/trehalose	5	36	2 : 3
**Proximity ligation assay (Fig. 3A, B, Supplementary Fig. 5)**				
MSA model mouse	Maltose/maltose	4	22.5	0 : 1
MSA model mouse	Trehalose/trehalose	3	32.3	0 : 1
**Filter trap assay TBS fraction (Fig. 3C, D, G, I)**				
MSA model mouse	Maltose/maltose	4	36	1:1
MSA model mouse	Trehalose/trehalose	4	36	1:1
**Filter trap assay Urea fraction (Fig. 3E, F)**				
MSA model mouse	Maltose/maltose	3	36	2:1
MSA model mouse	Trehalose/trehalose	3	36	2:1
We used same mouse as used in TBS fraction, but we prepared 3 samples in each group because of the amount of hippocampus.				
**Quantitative reverse transcription-polymerase chain reaction (Supplementary Fig. 4)**				
MSA model mouse	Maltose/maltose	3	22.67	1:2
MSA model mouse	Trehalose/trehalose	3	33.3	1:2

Abbreviations: MSA: multiple system atrophy. Some mice were used in multiple analyses.

Supplementary Table 2: Product information

Host	Antibody	Company	Product number	IH	WB	PLA
Rabbit	anti-Actin antibody	Sigma, St Louis	A5060		x10000	
Rabbit	Anti-LC3B antibody	Sigma	L7543		x4000	
Mouse	Anti-α-synuclein (syn211)	Abcam, Cambridge, UK	ab80627	x2000	x1000	
Mouse	Anti-α-synuclein (syn211), BSA and Azide free	Abcam	ab206675			20 µg
Rabbit	Anti-α-synuclein (phospho S129)	Abcam	ab51253	x2000	x2000	
Mouse	Anti-aggregated α-synuclein, clone 5G4	Merck Millipore, Burlington, MA	MABN389		x500	
Rabbit	α-Synuclein antibody	Cell Signaling Technology, Danvers, MA	#2642S		x4000	
Rabbit	Anti-Olig2 antibody [EPR2673]	Abcam	ab109186	x250		
Goat	Goat Anti-Rabbit IgG H&L (HRP)	Abcam	ab6721		x30000	
Goat	Goat Anti-Mouse IgG H&L (HRP)	Abcam	ab6789		x30000	
Goat	Goat Anti-Rabbit IgG Antibody (H+L), Biotinylated	Vector Laboratories, Burlingame, CA	BA-1000	x200		
Horse	Horse Anti-Mouse IgG Antibody (H+L), Biotinylated	Vector Laboratories	BA-2000	x200		

Product	Company	Product number
2-Deoxy-D-glucose	Fujifilm-wako, Osaka, Japan	040-06481
Maltose	Fujifilm-wako	137-00625
Normal goat serum	Vector Laboratories	S1000
Normal horse serum	Vector Laboratories	S2000
Recombinant human α-synuclein (wt) protein	Rpeptide, Watkinsville, GA	1001-1
Trehalose endotoxin free, MW=378.33	Fujifilm-wako	636-04121

Abbreviations: IH: Immunohistochemistry; WB: Western blot; PLA: Proximity ligation assay.

Figure 2H

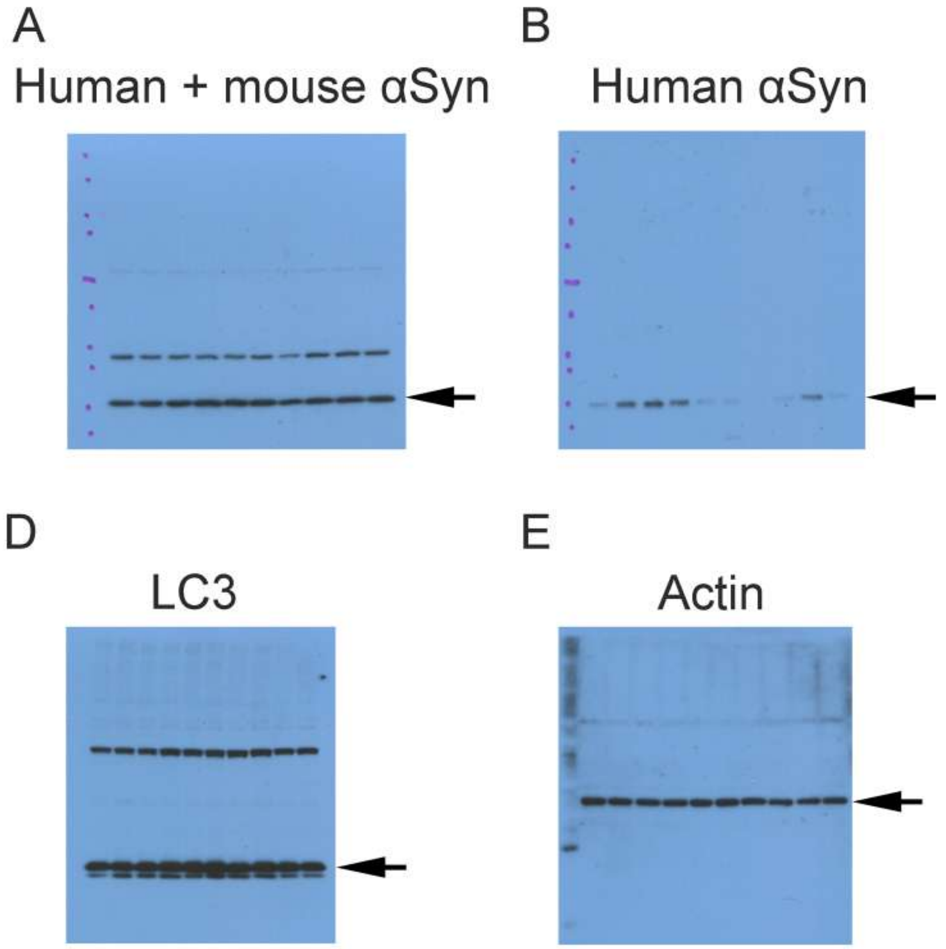

Figure 2M

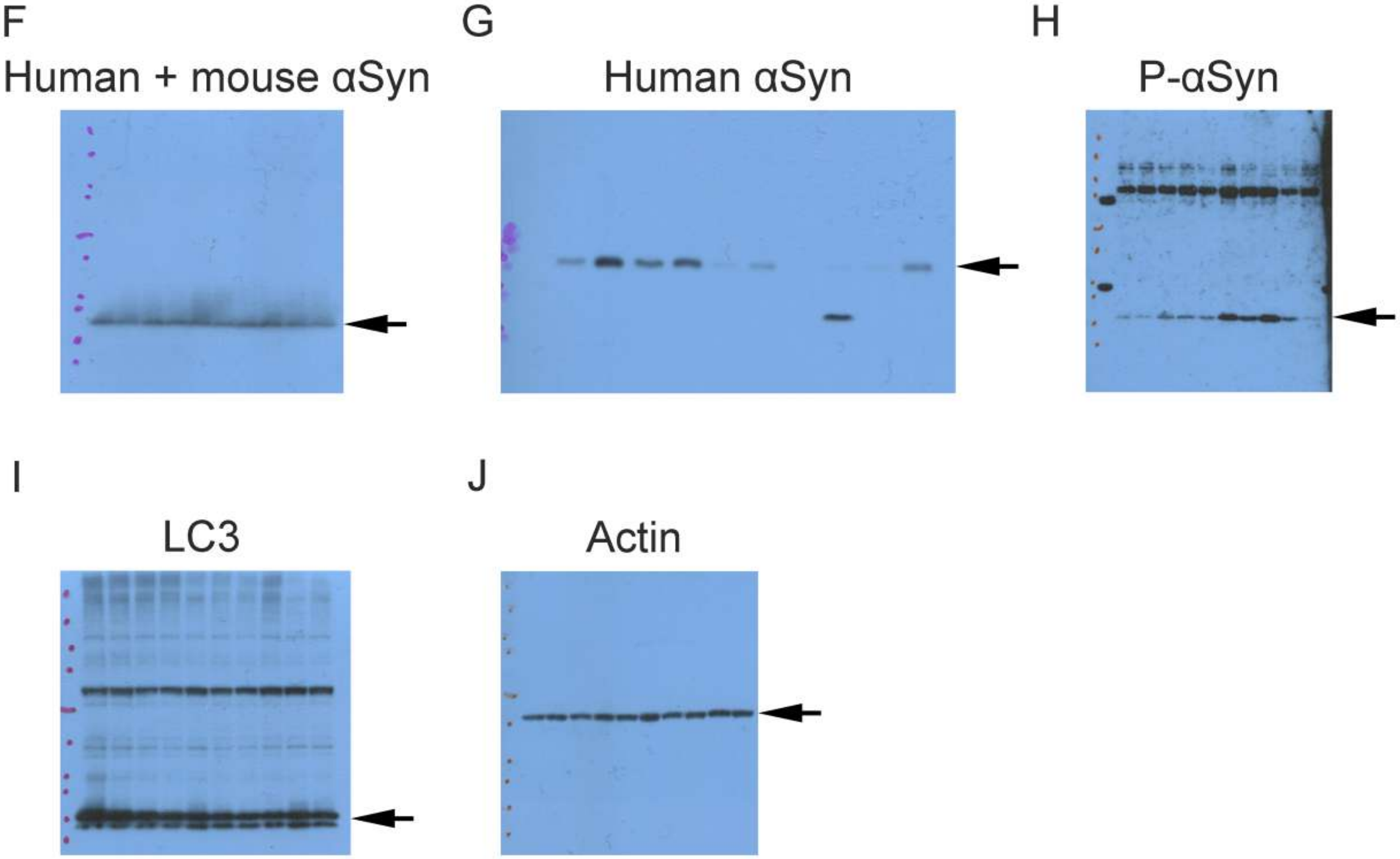

Figure 3C

K

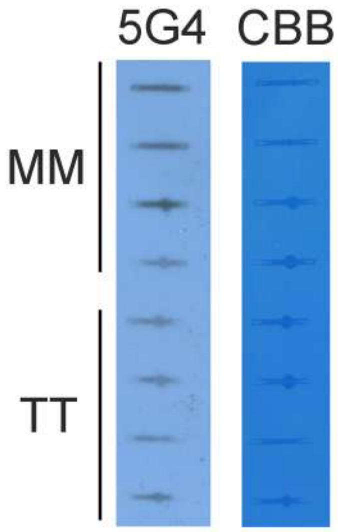

Figure 3E

L

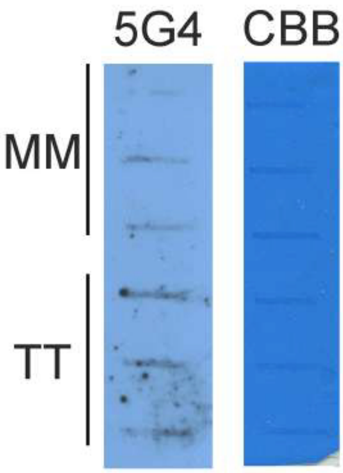

Figure 3G

M

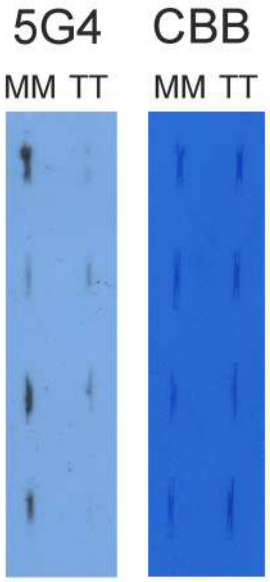

Figure 3I

N

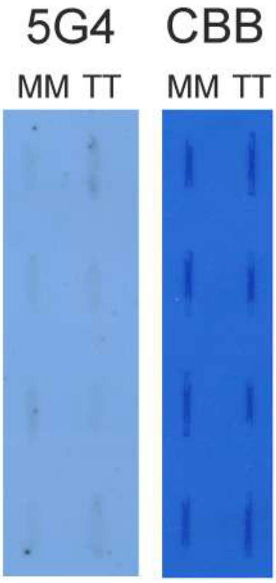

Supplementary Figure 6A

O

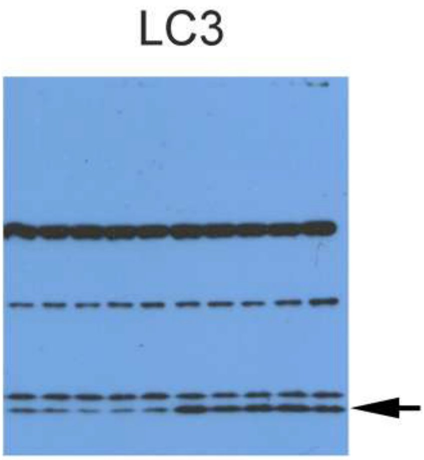

P

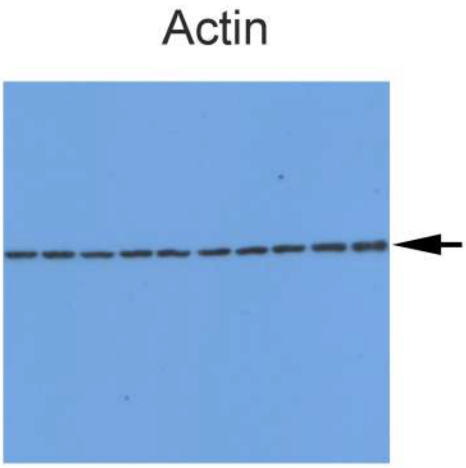

All uncropped results from western blotting analyses and filter trap assays. (A-E) The results used in Fig. 2H. (F-J) The results used in Fig. 2M. (K) The result used in Fig. 3C. (L) The result used in Fig. 3E. (M) The result used in Fig. 3G. (N) The result used in Fig. 3I. (O, P) The results used in Supplementary Figure 6A. The arrow indicates the bands we showed in figures.
